# Varus aligned patients have decreased patient reported International Knee Documentation Committee, knee injury and osteoarthritis outcome score sports scores and Tegner activity levels compared to neutral aligned patients in primary anterior cruciate ligament reconstructions

**DOI:** 10.1002/jeo2.70558

**Published:** 2025-11-14

**Authors:** Claire J. Knowlan, Luke V. Tollefson, Jace R. Otremba, Nicholas I. Kennedy, Christopher M. LaPrade, Robert F. LaPrade

**Affiliations:** ^1^ Creighton University School of Medicine Omaha Nebraska USA; ^2^ Twin Cities Orthopedics Edina Minnesota USA; ^3^ University of North Dakota School of Medicine and Health Sciences Grand Forks North Dakota USA; ^4^ Orthopedics Northwest Yakima Washington USA

**Keywords:** ACL tear, coronal malalignment, patient‐reported outcomes, posterior tibial slope, sagittal malalignment, valgus, varus

## Abstract

**Purpose/Hypothesis:**

The purpose was to evaluate the impact of coronal or sagittal alignment on patient‐reported outcomes after primary anterior cruciate ligament reconstruction (ACLR). The hypothesis was that coronal or sagittal alignment would not significantly influence 2‐year outcomes after a primary ACLR.

**Methods:**

Eighty patients with a mean age of 30.9 were included in this retrospective case series. Patients who underwent primary ACLR and at least 2 years of follow‐up completed the International Knee Documentation Committee survey, knee injury and osteoarthritis outcome score, Tegner activity level and the lysholm knee scoring scale. Scores were compared between varus, neutral and valgus alignments, as well as patients with a posterior tibial slope ≥12° or <12°. Independent two‐tailed *t*‐tests determined significant differences between the groups.

**Results:**

The primary outcome was patients with varus alignment had significantly decreased postoperative International Knee Documentation Committee survey (80.18) (*p* = 0.010), knee injury and osteoarthritis outcome score: Sports and rec (75.40) (*p* = 0.036) and Tegner activity level (6.00) (*p* = 0.002) scores compared to patients with neutral alignment (88.11, 85.37 and 7.61, respectively). No other groups showed a significant difference. A secondary outcome showed no significant difference between increasing coronal alignment deformity from neutral (180°) and patient‐reported outcomes.

**Conclusions:**

The primary outcome demonstrated significantly higher postoperative International Knee Documentation Committee Survey, knee injury and osteoarthritis outcome score: sports and recreation, and Tegner score in the neutral compared to the varus alignment groups, with knee injury and osteoarthritis outcome score: sports and recreation and Tegner score surpassing the minimally clinically important difference. No significant differences were observed between other coronal or sagittal groups. These findings suggest that varus alignment may be associated with clinically meaningful differences in patient outcomes compared to neutral alignment after primary ACLR.

**Level of Evidence:**

Level III, retrospective case series.

## Graphical abstract

This graphical abstract illustrates a comparison of patient‐reported outcome measures in patients undergoing primary anterior cruciate ligament reconstruction (ACLR). It compares outcomes across coronal and sagittal knee alignment groups. Patients with varus alignment demonstrated significantly lower International Knee Documentation Committee, knee injury and osteoarthritis outcome score, sports and recreation and Tegner activity scores compared to those with neutral alignment, while no other differences were observed in coronal or sagittal alignment. These findings suggest that native varus alignment may be associated with inferior patient‐reported outcomes following primary ACLR.

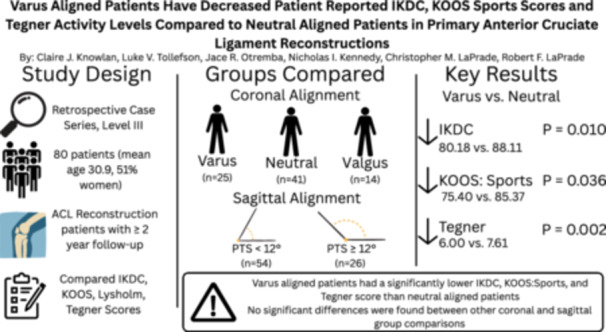

**Abbreviations**
ACL: anterior cruciate ligament
ACLR: anterior cruciate ligament reconstruction
BMI: body mass index
CI: confidence interval
FCL: fibular collateral ligament
HKA: hip‐knee‐ankle
IKDC: International Knee Documentation Committee
KOOS: knee injury and osteoarthritis outcome score
LET: lateral extra‐articular tenodesis
LTE: lateral tibial eminence
MCID: minimally clinically important difference
MCL: medial collateral ligament
MTE: medial tibial eminence
PCL: posterior cruciate ligament
PLC: posterolateral corner
POL: posterior oblique ligament
PROMs: patient‐reported outcome measures
PTR: patellar tendon reconstruction
PTS: posterior tibial slope

## INTRODUCTION

Anterior cruciate ligament (ACL) tears are among the most common knee injuries, and while reconstruction is typically successful, failures still occur, with reported failure rates ranging from 2.4% at 2 years to 5%–17% at over 10 years of follow‐up [5, 8, 17, 21, 29, 34]. The most frequently cited causes of ACL reconstruction (ACLR) failure include surgical technical error, particularly tunnel malposition, as well as unrecognised or untreated concomitant injuries such as meniscal or additional ligament tears [1, 11, 26].

Recently, studies have investigated how osseous malalignment can contribute to the ACLR failure rate. These studies have found that having an increased posterior tibial slope (PTS) (≥12°) can put more force on the ACL and thus increase the rate of ACL tears and graft failure [2, 3, 7, 14]. In addition, a relationship between ACLR failure and coronal malalignment has also been reported. It has been reported that in varus knees, the native ACL force significantly increases compared to neutrally aligned knees [32]. Studies have also reported that varus malalignment is correlated with increased load on the ACL and, hence, a higher risk of ACL tears and graft failure [13, 16, 19]. More recent studies have reported that having valgus malalignment also contributes to increased load on the ACL [18, 20, 23].

While increased valgus or varus alignment has been associated with higher stress on ACL grafts, no studies to the authors' knowledge have directly examined how coronal or sagittal malalignment affects patient‐reported outcome measures (PROMs) after primary ACLR. Addressing this relationship is important, as it may help guide surgical planning, such as the potential need for concomitant osteotomy to correct alignment, and individualised patient counselling to optimise functional outcomes.

The primary aim of this study was to evaluate if coronal or sagittal alignment is associated with differences in PROMs in patients at least 2 years out from a primary ACLR. Secondarily, this study aimed to determine if increasing degrees of coronal alignment was associated with decreased PROMs. The hypothesis was that coronal or sagittal alignment would not significantly influence 2‐year PROMs after a primary ACLR.

## METHODS

This is a retrospective cohort study and was approved by the institutional review board at BRANY (IRB# 23‐12‐443‐1135). It was performed on patients who had an ACLR performed by a single surgeon between July 2019 and December 2022 who had preoperative PROMs collected with a minimum of two years of postoperative follow‐up. Patients were excluded if they were younger than 15 years old at the time of surgery (as physeal closure and skeletal maturity occur after this age [6]), had concomitant osseous realignment surgery, had ACLR graft failure, or did not have preoperative PROMs. Patients undergoing additional ligament reconstruction (e.g., posterior cruciate ligament [PCL], medial collateral ligament [MCL], fibular collateral ligament [FCL]) or lateral extra‐articular tendinosis (LET) at the time of ACLR were included in this study. The presence and type of concomitant procedures were recorded and analysed in subgroup comparisons to assess their potential influence on PROMs. ACLR was defined as a first‐time ACL reconstruction with no prior ipsilateral ACL surgery, osteotomy, or realignment procedure. This research received no specific grant from any funding agency in the public, commercial, or not‐for‐profit sectors.

An anatomic single bundle ACLR with a bone‐patellar tendon‐bone autograft was performed in all patients. Patients were allowed weight bearing as tolerated except for those with a concurrent ligament tear or a radial or root meniscus tear where weight bearing was delayed until 6 weeks postoperatively. Patients followed a standardised postoperative rehabilitation program which involved strength, endurance and agility testing at 4, 7 and 10 months postoperatively. Patients were allowed to return to sports activities if they were at least 10 months postoperative and demonstrated a > 85% quadriceps limb symmetry index.

### Radiographic analysis

**Figure 1:**
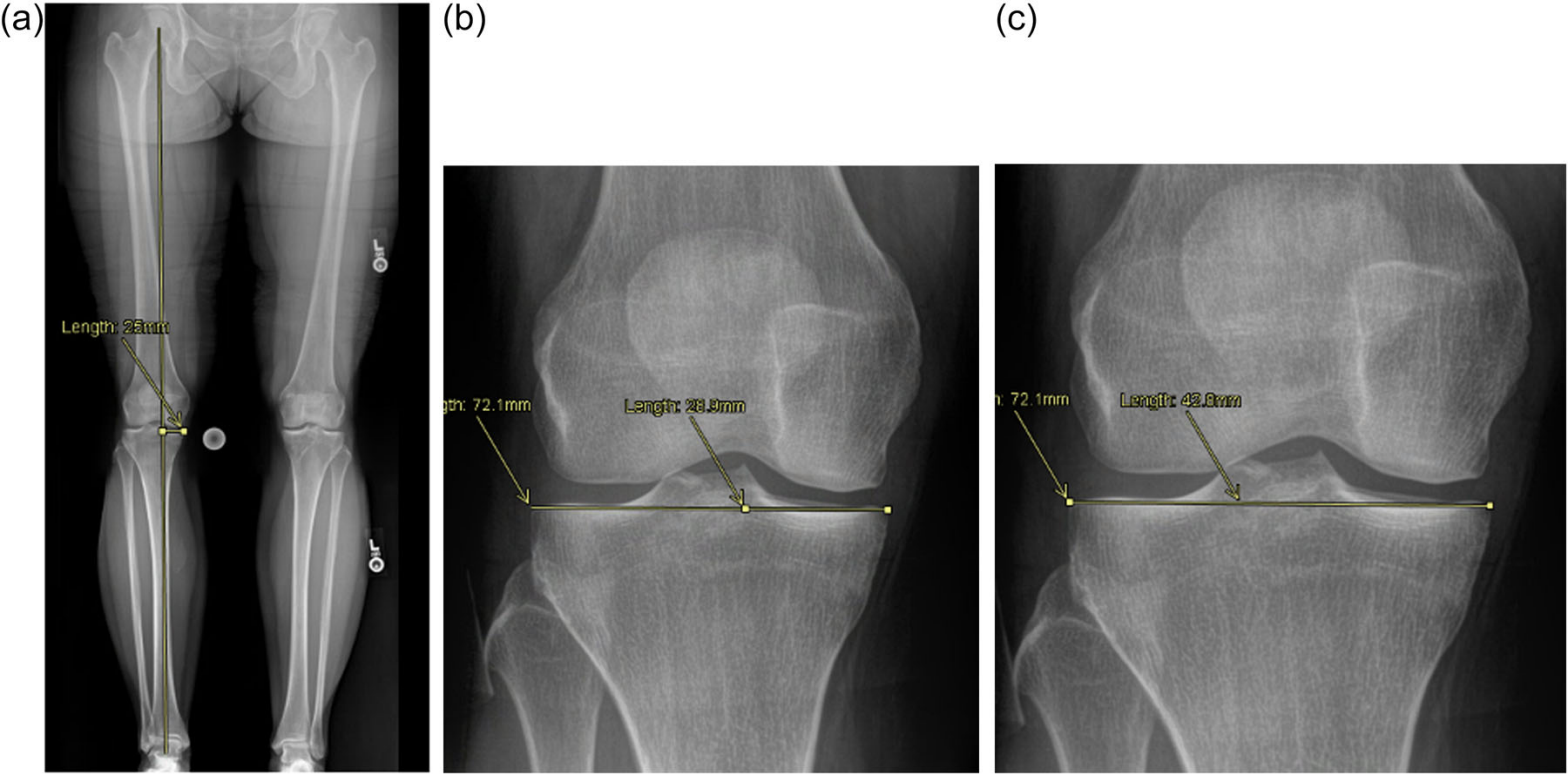
Summary of radiographic measurements performed to determine patient coronal alignment: (a) Distance from the medial tibial plateau to the dropline. (b) Distance from the medial tibial plateau to the MTE. (c) Distance from the medial tibial plateau to the LTE. LTE, lateral tibial eminence; MTE, medial tibial eminence.

During the clinical diagnostic work‐up for all patients with suspected ACL tears, radiographs were obtained to assess the injury and the patient's native coronal and sagittal alignment. These measurements were performed by two medical student research interns working under the operating surgeon. Researchers were not blinded to patient identifiers but were blinded to each other's measurements, as they were measured independently. Standardised weight‐bearing positioning was used when collecting radiographs. To assess coronal alignment, bilateral long‐leg anteroposterior standing view images were obtained. To establish varus, valgus, or neutral alignment, a digital line was drawn from the centre of the femoral head to the centre of the tibiotalar joint to determine the long leg weightbearing axis (dropline). If the dropline was between the medial tibial eminence (MTE) and lateral tibial eminence (LTE), the patient had neutral alignment. If the dropline was medial to the MTE, the patient had varus alignment. Finally, if the dropline was lateral to the LTE, the patient had valgus alignment. An example of these measurements can be seen in Figure 1. The degree of varus or valgus alignment was based on the hip‐knee‐ankle (HKA) angle [12].

**Figure 2:**
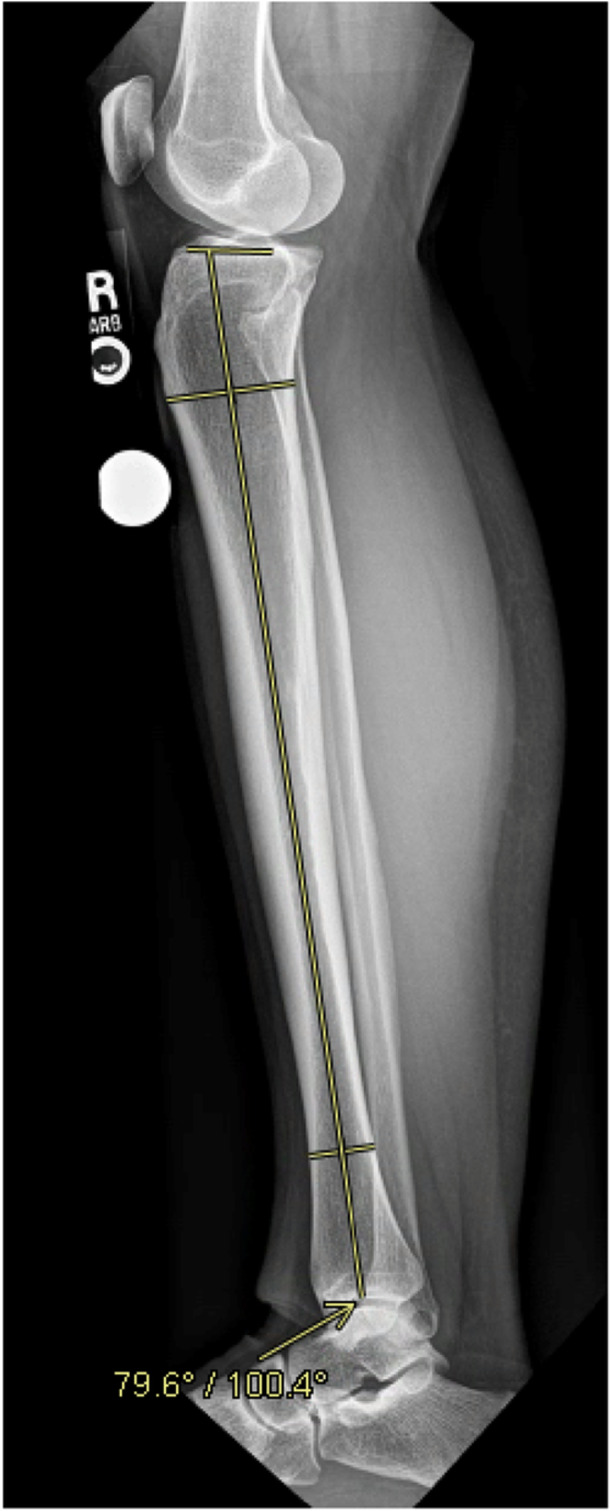
Summary of the radiographic measurement performed to determine the PTS (10.4° in this example). The PTS is the angle between the lateral tibial plateau and tibial anatomic axis. The tibial anatomic axis was found using a mid‐diaphyseal line, which was centred using two perpendicular lines (not shown) spanning the width of the tibia. The PTS was the angle found subtracted from 90°. PTS, posterior tibial slope.

To determine sagittal alignment, PTS was measured using lateral radiographs. It was measured as the angle between the lateral tibial plateau and the tibial anatomic axis. The tibial anatomic axis was found using a mid‐diaphyseal line, which was centred on the tibia using two perpendicular lines spanning the width of the tibia 5 cm distal to the tibiofemoral joint line and 5 cm proximal to the tibiotalar joint line [2]. A threshold of 12° was selected based on previously published data linking increased slope to higher ACL graft strain and failure rates [2, 7, 10, 14]. An example of this measurement can be seen in Figure 2.

### PROMs

This study protocol required obtaining PROMs questionnaire responses from patients who underwent surgery and had at least 2 years of follow‐up. PROMs included the International Knee Documentation Committee (IKDC) [15], Tegner activity score [31], lysholm knee scoring scale [22] and the knee injury and osteoarthritis outcome score (KOOS) [27]. Patients' scores on these questionnaires were then calculated to assess their reported outcomes. Scores were analysed as continuous variables (mean ± standard deviation).

The primary outcome was the difference in postoperative PROMs between coronal alignment groups at the 2‐year postoperative time point. The primary outcome was comparative in nature, assessing whether alignment influenced PROMs. Secondary outcomes included: Differences in postoperative PROMs between sagittal alignment groups, changes in PROMs from preoperative to postoperative assessments, correlations between the degree of coronal alignment and PROMs, and differences in PROMs based on concomitant procedures, including LET, ligament reconstructions and meniscal procedures.

### Minimally clinically important differences (MCID)

Once PROMs were collected by category, any group showing a statistically significant difference from another was evaluated against literature‐based MCID values specific to the ACLR population. This was done to see if a clinically significant difference would be expected. The literature values can be seen in Table 1.

**Table 1:** Literature‐based MCID values by category, specific to the ACLR population.

	IKDC [25]	Lysholm [25]	KOOS: Daily living [28]	KOOS: Pain [28]	KOOS: Quality of life [28]	KOOS: Sports and rec [28]	Tegner activity level [33]
MCID	9.5	10.6	8.0	6.1	7.2	5.8	1

Abbreviations: ACLR, anterior cruciate ligament reconstruction; IKDC, International Knee Documentation Committee; KOOS, knee injury and osteoarthritis outcome score; Lysholm, lysholm knee scoring scale; MCID, minimally clinically important differences.

### Statistical analysis

After receiving the PROMs, patients were grouped by coronal alignment (varus, neutral, valgus) and sagittal alignment (PTS ≥ 12° vs. PTS < 12°). The primary outcome of comparing differences between postoperative PROMs between coronal alignment groups was analysed using independent samples two‐tailed *t*‐tests. Statistical significance defined as *p*‐value ≤ 0.05. ≥0.9 interclass correlation coefficient (ICC) was required to proceed with the study. Results were reported at the mean score ± standard deviation and *p*‐values. Comparisons included: varus versus neutral, valgus versus neutral and varus versus valgus for coronal alignment. A secondary outcome was a postoperative comparison of PROMs for PTS ≥ 12° versus PTS < 12°.

The data were arranged in order of increasing deviation from 180°, as determined by the absolute value of the HKA angle. Each PROM was then analysed against the absolute value of increasing deviation from neutral (180°—HKA angle) to assess if increasing malalignment was associated with differences in PROMs. Significance of this data was determined with Pearson correlation coefficients and associated *p*‐values. This analysis was performed in Python (v3.11) using the pandas, scipy, and statsmodels packages.

Given this is a retrospective study, an a priori sample size calculation was not feasible. Therefore, a post hoc power analysis was conducted to determine whether the study had sufficient power to detect observed effect sizes. This was conducted via Python (v3.11) with the statsmodels and pingouin packages, for each outcome to determine the likelihood of detecting a true effect given the observed effect and sample sizes. Independent samples two‐tailed *t*‐tests were initially conducted to compare outcomes between the coronal groups and the sagittal group. For each comparison, Cohen's *d* was determined to estimate the standardised effect size for each comparison. Power was then calculated using these effect sizes, with significance set at *α* = 0.05.

## RESULTS

**Figure 3:**
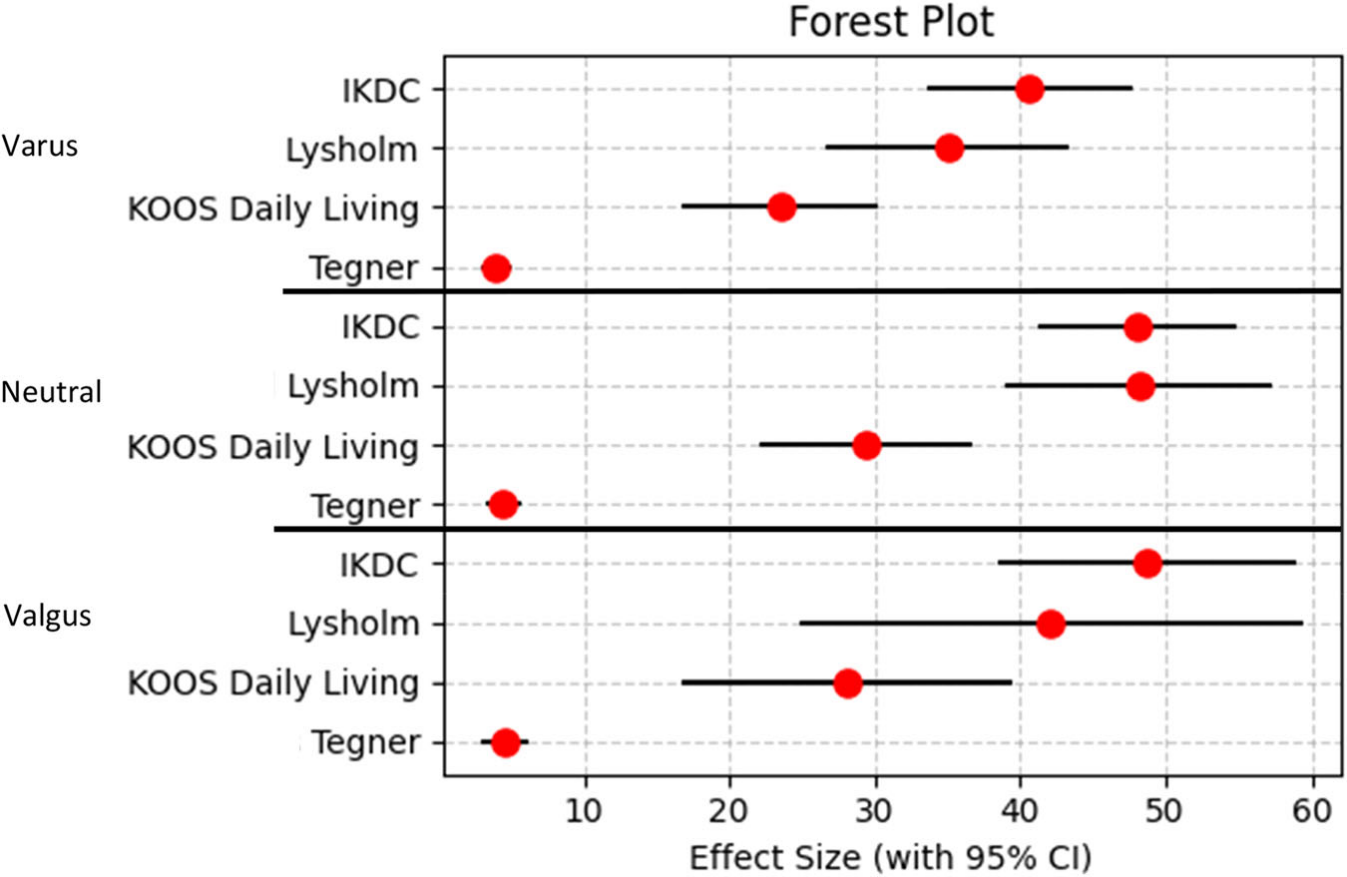
Forest plot displaying the mean changes from preoperative to postoperative PROMs with 95% CIs for coronal alignment. PROMs included are IKDC, Lysholm, KOOS: Daily Living, and Tegner. The red dots represent the averages, while the horizontal lines indicate the 95% CIs. CI, confidence interval; IKDC, International Knee Documentation Committee; KOOS, knee injury and osteoarthritis outcome score; Lysholm, lysholm knee scoring scale; PROM, patient‐reported outcome measures.

In total, 100 patients were eligible to participate in this study. Twenty patients were lost to follow‐up, amounting to a total of 80 participants (80% follow‐up). The average follow‐up time for participants in this study was 31.85 ± 6.90 months. Included in this study were 39 men and 41 women and an average age of 30.9 years old (range, 15–64 years). All PROMs showed significant improvement from preoperative to postoperative scores (*p* < 0.05). The average preoperative and postoperative scores for the PROMs can be seen in Table 2. Mean changes between preoperative and postoperative scores with 95% confidence intervals (CIS) are illustrated in Figure 3.

**Table 2:** Average preoperative and postoperative PROMs for each of the coronal and sagittal alignments after primary ACLR.

	IKDC	Lysholm	KOOS: Daily living	KOOS: Pain	KOOS: Quality of life	KOOS: Sports	Tegner activity level
Varus							
Pre‐Op	39.54 ± 11.88 (34.64–44.44)	54.60 ± 19.11 (46.71–62.49)	72.82 ± 17.13 (65.75–79.89)	69.60 ± 15.16 (63.34–75.86)	27.25 ± 20.88 (18.63–35.87)	19.60 ± 25.16 (9.21–29.99)	2.04 ± 0.93 (1.65–2.43)
Post‐Op	80.18 ± 14.22 (74.31–86.05)	89.64 ± 9.46 (85.74–93.55)	96.29 ± 5.65 (93.96–98.63)	88.78 ± 11.74 (83.93–93.62)	73.25 ± 19.64 (65.14–81.36)	75.40 ± 23.18 (65.83–84.97)	6.00 ± 2.06 (5.15–6.85)
Neutral							
Pre‐Op	40.08 ± 19.00 (34.40–45.77)	44.32 ± 25.65 (36.22–52.41)	67.00 ± 22.26 (59.98–74.03)	62.60 ± 16.49 (57.39–67.81)	19.97 ± 17.63 (14.40–25.54)	23.90 ± 24.91 (16.04–31.77)	3.17 ± 2.78 (2.29–4.05)
Post‐Op	88.11 ± 10.05 (84.94–91.29)	92.46 ± 7.81 (90.00–94.93)	96.38 ± 8.00 (93.85–98.90)	92.82 ± 8.45 (90.15–95.48)	77.44 ± 19.56 (71.27–83.61)	85.37 ± 14.64 (80.75–89.98)	7.61 ± 1.86 (7.02–8.20)
Valgus							
Pre‐Op	38.18 ± 14.54 (29.78–46.57)	49.00 ± 31.04 (31.08–66.92)	68.58 ± 21.44 (56.20–80.96)	61.64 ± 18.00 (51.25–72.03	22.29 ± 23.67 (8.62–35.95)	30.36 ± 28.11 (14.13–46.59)	2.36 ± 1.50 (1.49–3.22)
Post‐Op	86.86 ± 10.18 (80.99–92.73)	91.07 ± 10.64 (84.93–97.21)	96.70 ± 5.10 (93.75–99.64)	93.65 ± 6.66 (89.80–97.50)	80.36 ± 17.31 (70.36–90.35)	83.21 ± 18.97 (72.26–94.17)	6.93 ± 1.69 (5.96–7.90)
Slope ≥ 12°							
Pre‐Op	40.23 ± 15.23 (34.08–46.38)	47.92 ± 24.05 (38.21–57.64)	70.98 ± 18.25 (63.60–78.35)	67.31 ± 16.76 (60.54–74.08)	29.31 ± 25.15 (19.16–39.46)	23.27 ± 27.60 (12.12–34.42)	2.85 ± 2.48 (1.85–3.85)
Post‐Op	87.09 ± 10.13 (83.00–91.18)	92.69 ± 9.08 (89.02–96.36)	97.20 ± 4.23 (95.50–98.91)	93.16 ± 7.67 (90.07–96.26)	79.09 ± 17.94 (71.84–86.33)	85.19 ± 15.20 (79.05–91.33)	6.85 ± 2.11 (5.99–7.70)
Slope < 12°							
Pre‐Op	39.27 ± 15.85 (34.94–43.59)	48.56 ± 25.62 (41.56–55.55)	68.19 ± 21.67 (62.28–74.11)	63.32 ± 16.38 (58.85–67.79)	19.44 ± 15.95 (15.10–23.80)	23.89 ± 24.68 (17.15–30.63)	2.59 ± 2.07 (2.03–3.16)
Post‐Op	84.61 ± 12.72 (81.14–88.08)	90.69 ± 8.75 (88.30–93.07)	96.02 ± 7.76 (93.91–98.14)	91.00 ± 10.19 (88.21–93.78)	75.46 ± 19.76 (70.07–80.86)	80.28 ± 20.15 (74.78–85.78)	7.06 ± 1.97 (6.52–7.59)

*Note*: Reported as mean ± standard deviation (95% CI).

Abbreviations: ACLR, anterior cruciate ligament reconstruction; CI, confidence interval; IKDC, International Knee Documentation Committee; KOOS, knee injury and osteoarthritis outcome score; Lysholm, lysholm knee scoring scale; PROM, patient‐reported outcome measures.

### Coronal alignment

Of the 80 patients included in the study, 41 had neutral alignment, 25 had varus alignment, and 14 had valgus alignment. Demographics for coronal alignment, as well as the average HKA angle and PTS can be seen in Table 3.

**Table 3:** Demographics, average HKA angle and average PTS of coronal alignment groups after primary ACLR.

	Varus	Neutral	Valgus
Sex			
Males (*n*)	13	23	3
Females (*n*)	12	18	11
Age	33.72 (27.33–40.11)	29.49 (25.38–33.60)	30.64 (20.99–40.29)
BMI	25.45 (23.79–27.11)	25.65 (24.11–27.19)	24.09 (21.97–26.21)
HKA angle	177.06 (176.42–177.70)	180.02 (179.59–180.46)	182.96 (182.09–183.83)
PTS	10.52 (9.18–11.86)	10.33 (9.41–11.25)	10.24 (8.62–11.86)

*Note*: Data reported as a number (*n*) or as the mean (95% CI).

Abbreviations: ACLR, anterior cruciate ligament reconstruction; BMI, body mass index; CI, confidence interval; HKA, hip‐knee‐ankle; PTS, posterior tibial slope.

The primary outcome of this study showed there was a significant decrease in the IKDC (*p* = 0.010), KOOS: sports and rec (*p* = 0.036) and Tegner activity level (*p* = 0.002) scores for patients with varus alignment compared to those with neutral alignment. There were no significant differences between any other groups (Table 4). Among these, only the KOOS: Sports and Rec and Tegner scores exceed established MCID thresholds, suggesting clinical relevance. The difference in IKDC scores, while statistically significant, did not meet the MCID and may not reflect a meaningful clinical distinction.

**Table 4:** *p* values comparing each form of coronal plane alignment to PROMs after primary ACLR.

	IKDC	Lysholm	KOOS: Daily Living	KOOS: Pain	KOOS: Quality of life	KOOS: Sports	Tegner activity level
Varus versus valgus	0.131	0.667	0.997	0.163	0.266	0.290	0.159
Varus versus neutral	0.010	0.194	0.964	0.110	0.403	0.036	0.002
Neutral versus valgus	0.691	0.603	0.978	0.830	0.700	0.662	0.231

Abbreviations: ACLR, anterior cruciate ligament reconstruction; IKDC, International Knee Documentation Committee; KOOS, knee injury and osteoarthritis outcome score; Lysholm, lysholm knee scoring scale; PROM, patient‐reported outcome measures.

There was no significant relationship between PROMs and increasing deviation from neutral (180°—HKA angle), as indicated by nonsignificant Pearson correlation coefficients and associated *p*‐values. The patient with the largest deviation from neutral was in varus alignment and had a difference from neutral (180°) of 7.5°.

### Sagittal alignment

Demographics for sagittal alignment, the average HKA angle, and PTS can be seen in Table 5. For sagittal alignment, there was no significant difference between PROMs for PTS ≥ 12° and PTS < 12° (Table 6). When comparing the LET patients to non‐LET patients, it was found that there was a significant difference (*p* = 0.0365) between the groups for the postoperative Tegner scores. Patients with a LET had an average Tegner score of 8 and those without an LET had an average Tegner score of 6.77. None of the other PROMs had significant differences between the LET and non‐LET groups.

**Table 5:** Demographics, average HKA angle, average PTS, and concomitant LET in sagittal alignment groups post‐ACLR.

	Slope ≥ 12°	Slope < 12°
Sex		
Males (*n*)	15	24
Females (*n*)	11	30
Age	29.69 (24.32–35.06)	31.24 (27.15–35.33)
BMI	25.71 (24.09–27.33)	25.10 (23.84–26.36)
HKA angle	179.24 (177.95–180.53)	179.79 (179.22–180.36)
PTS	13.72 (13.28–14.16)	8.76 (8.19–9.33)
LET		
With LET (*n*)	4	10
Without LET (*n*)	22	44

*Note*: Data are reported as either a number (*n*) or as the mean (95% CI).

Abbreviations: ACLR, anterior cruciate ligament reconstruction; BMI, body mass index; CI, confidence interval; HKA, hip‐knee‐ankle; LET, lateral extra‐articular tenodesis; PTS, posterior tibial slope.

**Table 6:** *p* values comparing each form of sagittal plane alignment to postoperative PROMs after primary ACLR.

	IKDC	Lysholm	KOOS: Daily Living	KOOS: Pain	KOOS: Quality of life	KOOS: Sports	Tegner activity level
Slope ≥ 12° versus slope < 12°	0.387	0.345	0.471	0.341	0.431	0.274	0.664

Abbreviations: ACLR, anterior cruciate ligament reconstruction; IKDC, International Knee Documentation Committee; KOOS, knee injury and osteoarthritis outcome score; Lysholm, lysholm knee scoring scale; PROM, patient‐reported outcome measures.

### Concomitant ligament injuries

Thirty‐six patients (45%) had a concomitant ligament reconstruction during their ACLRs, including 6 PCL reconstructions (7.5%), 13 MCL reconstructions (16.3%), 22 FCL reconstructions (27.5%), 1 posterolateral corner (PLC) reconstruction (1.3%) and 1 posterior oblique ligament (POL) reconstruction (1.3%). In all the PROMs, there were no significant differences in the outcomes between patients undergoing ACLR and those undergoing multiligamentous reconstruction. Also in this study, 70 of the 80 patients (87.5%) had some form of meniscal injury, including root tears, radial tears, bucket handle tears, ramp tears and vertical tears. Twenty‐two patients with varus alignment had meniscal injuries (88%), 36 patients with neutral alignment had meniscal injuries (87.8%) and 12 patients in the valgus group had meniscal injuries (85.7%). There were no significant differences in PROMs between patients who has meniscal injuries compared to those who did not.

### Post hoc power analysis

A post hoc power analysis was done for the different categories (Table 7). The highest‐powered groups were IKDC (0.742), KOOS: sports (0.561) and Tegner activity level (0.898) in the varus versus neutral category.

**Table 7:** Calculated post hoc power values for group comparisons for coronal alignment and posterior tibial slope across PROMs.

	IKDC	Lysholm	KOOS: Daily living	KOOS: Pain	KOOS: Quality of life	KOOS: Sports	Tegner activity level
Varus versus valgus	0.325	0.071	0.055	0.284	0.196	0.182	0.288
Varus versus neutral	0.742	0.253	0.050	0.359	0.132	0.561	0.898
Neutral versus valgus	0.068	0.081	0.052	0.062	0.078	0.072	0.221
Slope ≥ 12° versus slope < 12°	0.138	0.155	0.110	0.157	0.122	0.193	0.071

Abbreviations: IKDC, International Knee Documentation Committee; KOOS, knee injury and osteoarthritis outcome score; Lysholm, lysholm knee scoring scale; PROM, patient‐reported outcome measures.

### Complications

Five patients underwent a postoperative knee manipulation with an arthroscopic lysis of adhesions due to stiffness (5.3%). One patient had a seroma that resolved (1.3%). One patient had a patellar tendon reconstruction (PTR) concomitantly with their ACLR due to a patellar tendon rupture, but the PTR failed, so a revision PTR needed to be performed but the ACLR remained stable and functional (1.3%).

## DISCUSSION

The main finding of this study was that there was a significant decrease in the IKDC scores, KOOS: Sports and Rec scores, and Tegner activity level scores for patients who underwent a primary ACLR with varus alignment compared to the patients with neutral alignment. However, there were no significant differences in PROMs between valgus and neutral, and valgus and varus alignments. Additionally, no significant relationship was found between the degree of coronal malalignment and PROMs. Furthermore, in the primary ACL tear setting, there was no difference in PROMs between those with a slope ≥12° and those with a slope <12°. These results should be interpreted as exploratory and not definitive, given the small sample size and retrospective nature of the study. It is important to mention that although patients undergoing multiligament reconstruction were included in this study, no significant differences in PROMs were observed; however, the potential impact of surgical complexity warrants further future study.

When comparing preoperative PROMs to postoperative PROMs, every category improved by an amount greater than the MCID reported in the literature (Table 1) [25, 28, 33]. Among the postoperative comparisons between neutral and varus alignment, only the KOOS: Sports and rec (9.22) and Tegner (1.49) scores exceeded the MCID, suggesting clinically meaningful differences in these domains. In comparison, the IKDC scores did not surpass the literature‐defined MCID. This suggests that while a statistically significant difference may exist between neutral and varus alignment IKDC scores, this may not translate into being clinically noticeable for patients. This highlights the distinction that statistical significance does not equate to clinical significance. Since not all PROMs reached statistical or clinical significance, these findings alone do not warrant changes to current treatment protocols. Rather, they may inform patient counselling regarding expected outcomes in varus‐aligned knees.

As noted, PROMs were significantly lower for the varus group compared to the neutral group for IKDC score, KOOS: Sports and Rec score and Tegner Activity Level, but not significantly different between any of the other groups undergoing a primary ACLR. These findings are partly congruent with Kim et al., who reported that when a patient has varus alignment and no varus thrust, performing an ACLR without correction of malalignment results in maintenance of stability and high function [19]. In contrast with our results which showed no difference between valgus and neutral alignment, a study by Mehl et al. using cadaveric legs reported that the knees had decreased instability when an osteotomy was performed in conjunction with an ACLR for valgus‐aligned knees. With this in mind, performing a corrective osteotomy for severe valgus alignment (HKA ≥ 190°) may be considered when reconstructing the ACL, but should likely be reserved for ACL revisions [23]. Taken together, these previous studies demonstrated that there is no consensus on the optimal method of treatment for patients that have coronal malalignment and ACL tears, and treatment could vary between the degree of malalignment.

It is important to note that this study evaluated patients who underwent a primary ACLR, and patients who underwent a revision ACLR were excluded. In patients undergoing a revision ACLR, malalignment should be considered as a source of graft failure and corrected if found to be a primary cause of failure [9]. While this study did not identify a significant difference between PROMs and increasing degrees of varus or valgus alignment, biomechanical studies have reported that greater malalignment in both the coronal and sagittal planes leads to more stress on the ACL [2, 3, 7, 13, 14, 16, 18, 19, 23]. For example, van de Pol et al. showed in varus knees, mild varus alignment does not yield significant ACL tension, however, severe varus alignment (HKA < 170°) can create excessive tension on the ACL which can lead to graft failure [16]. The lack of significance associated between increasing varus and valgus alignment and poor PROMs in this study could be due to a limited number of patients with extreme varus or valgus alignment. Future studies should continue to evaluate how significant varus or valgus, especially more than 5 degrees, contributes to ACL graft forces and knee kinematics.

The findings from this study report that increased slope, specifically PTS ≥ 12° versus a PTS < 12°, for primary ACLR, had no significant differences in PROMs. This contrasts previous studies which have previously reported that there is a higher graft failure rate when the tibial slope is ≥12° [2, 3, 7, 10, 14]. A study by Ni et al. reported that excessive PTS (≥17°) is a predictive factor of primary ACLR failure, whereas demographics and concomitant meniscal tears were not predictive factors of failure [24]. Similarly, Bosco et al. reported that an anterior closing wedge high tibial osteotomy is a viable option to protect the reconstructed ACL and restore knee stability in both primary ACLR and revision ACLR in patients with high PTS [4]. In a study conducted by Song et al., results similar to those of Bosco et al. were found, and it was determined that performing a slope‐reducing osteotomy can enhance knee stability when performed with a concomitant ACLR [30]. While the results from this study diverge from these previous reports by showing no difference in PROMs for sagittal alignment, this reflects that PROMs capture a patient's subjective outcome rather than graft loading and stability. Thus, while a high PTS may predispose a patient to increased risk of failure, this may not be evident in PROMs.

To the authors' knowledge, this is the first study to evaluate how both coronal and sagittal alignment independently affect patient‐reported outcomes following primary ACL reconstruction. The study provides novel insight into this relationship and interprets findings in the context of established MCID thresholds, enhancing the clinical applicability and relevance of the results. This study has several limitations that should be acknowledged. A higher Tegner score was observed among patients who underwent LET. However, LET was typically reserved for patients with high‐grade pivot shift, ligamentous laxity, or those engaged in high‐level sports. Thus, this may introduce selection bias, as these patients may have had a higher preoperative activity level and motivations for return to sport, which may confound interpretation of the observed differences. Many patients had concomitant multiligamentous injuries and different baseline activity levels, both of which may have influenced pre‐ and postoperative outcomes. The sample was small and uneven across groups, with the varus group older than the neutral group, potentially affecting IKDC, KOOS and Tegner scores. Additionally, we acknowledge that our cohort was older compared to prior reports. This may be related to referral patterns at our institution, regional demographic differences, or the possibility that older patients were more likely to undergo surgery during the study period. Sagittal alignment was presented as a binary cutoff of 12° and not continuous, which may have reduced statistical sensitivity. Data analysis was done via multiple independent t‐tests across different categories without correction for multiplicity. This increases the risk for Type I error in the study. The study was not powered a priori to detect minimally clinically important differences, and small or unequal group sizes, particularly in the valgus cohort, may increase the risk of Type II error. Finally, coronal and sagittal alignment measurements are subject to inter‐ and intraobserver variability, which was minimised by having two investigators independently measure the radiographs.

Although this study did reveal some notable preliminary findings—specifically the differences in PROMs between the varus and neutral aligned groups—no definitive clinical recommendations can be made from this data alone. Instead, it is recommended that this data be used preoperatively to guide patient discussions regarding expected outcomes in varus‐aligned knees. This data should not be interpreted as definitive, but rather exploratory, serving as a foundation for future studies. A larger, prospective cohort study with balanced group sizes and an appropriate power analysis is needed to confirm these findings and guide future clinical decision making.

## CONCLUSIONS

The primary outcome demonstrated significantly higher postoperative International Knee Documentation Committee survey, knee injury and osteoarthritis outcome score: sports and recreation, and Tegner score in the neutral compared to the varus alignment groups, with knee injury and osteoarthritis outcome score: sports and recreation and Tegner score surpassing the minimally clinically important difference. No significant differences were observed between other coronal or sagittal groups. These findings suggest that varus alignment may be associated with clinically meaningful differences in patient outcomes compared to neutral alignment after primary ACLR.

## AUTHOR CONTRIBUTIONS

All authors contributed to the study design and concept. Claire Knowlan contacted patients, collected and analysed the data and drafted the main version of the manuscript. Luke Tollefson determined patient eligibility, performed patient alignment measurements, categorised the patients based on alignment, and contributed to manuscript revisions and guidance. Jace Otremba assisted in contacting patient to collect data, helped draft the main manuscript and provided ongoing editorial input and revisions. Nicholas Kennedy conceived the initial study idea and contributed to manuscript revisions. Christopher LaPrade provided editorial feedback and study guidance. Robert LaPrade contributed to manuscript revisions and guidance and served as the corresponding author. All authors read and approved the final version of the manuscript.

## CONFLICT OF INTEREST STATEMENT

Dr. Robert F. LaPrade is a consultant for Ossur and Smith and Nephew, receives royalties from Elsevier, Ossur and Smith and Nephew and has received research grants from AANA, AOSSM, Ossur and Smith and Nephew. The remaining authors declare no conflicts of interest.

## ETHICS STATEMENT

This retrospective case series involving human subjects complied with the ethical standards of the institutional and national research committees. This study was approved by the institutional review board at BRANY (IRB# 23‐12‐443‐1135). Written informed consent was obtained from all patients included in this study.

## Data Availability

The data that support the findings of this study are available from the corresponding author upon reasonable request.
